# Supplementary figures and images for: A novel model based on clinical and computed tomography (CT) indices to predict the risk factors of postoperative major complications in patients undergoing pancreaticoduodenectomy
Source: PeerJ. 2024 Dec 19;12:e18753. doi: 10.7717/peerj.18753 (PMC11663404; doi:10.7717/peerj.18753)

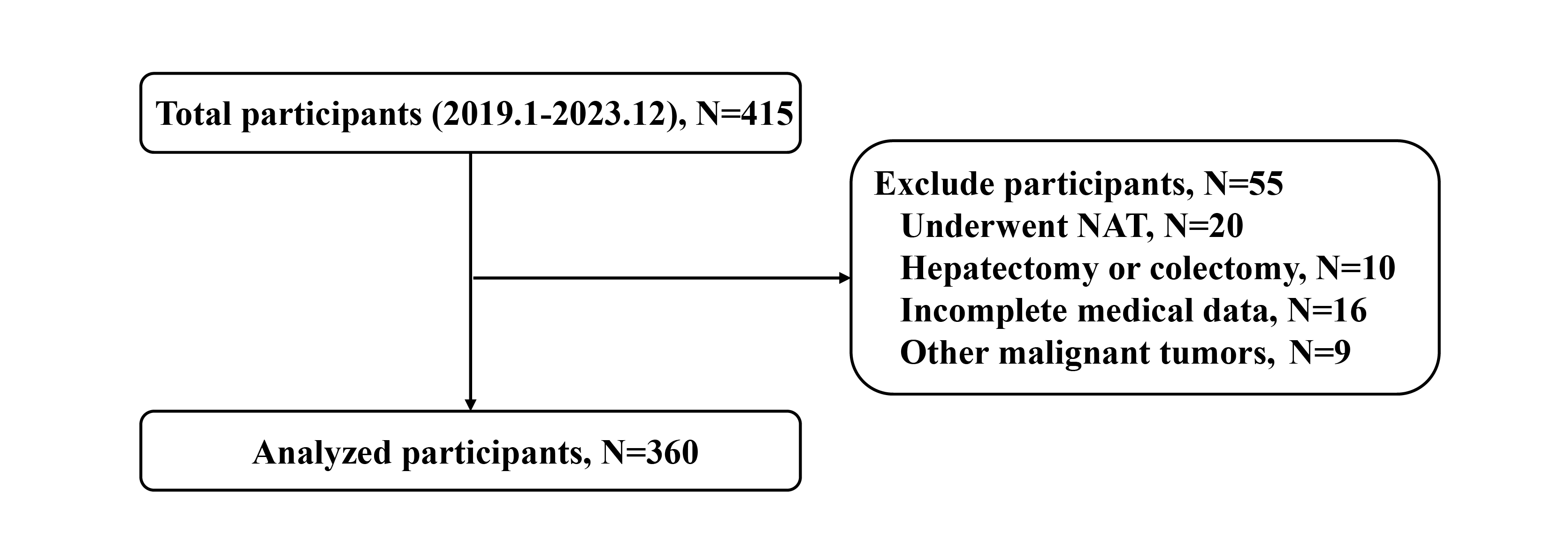

Supplement: Supplemental Information 1 [file peerj-12-18753-s001.png]

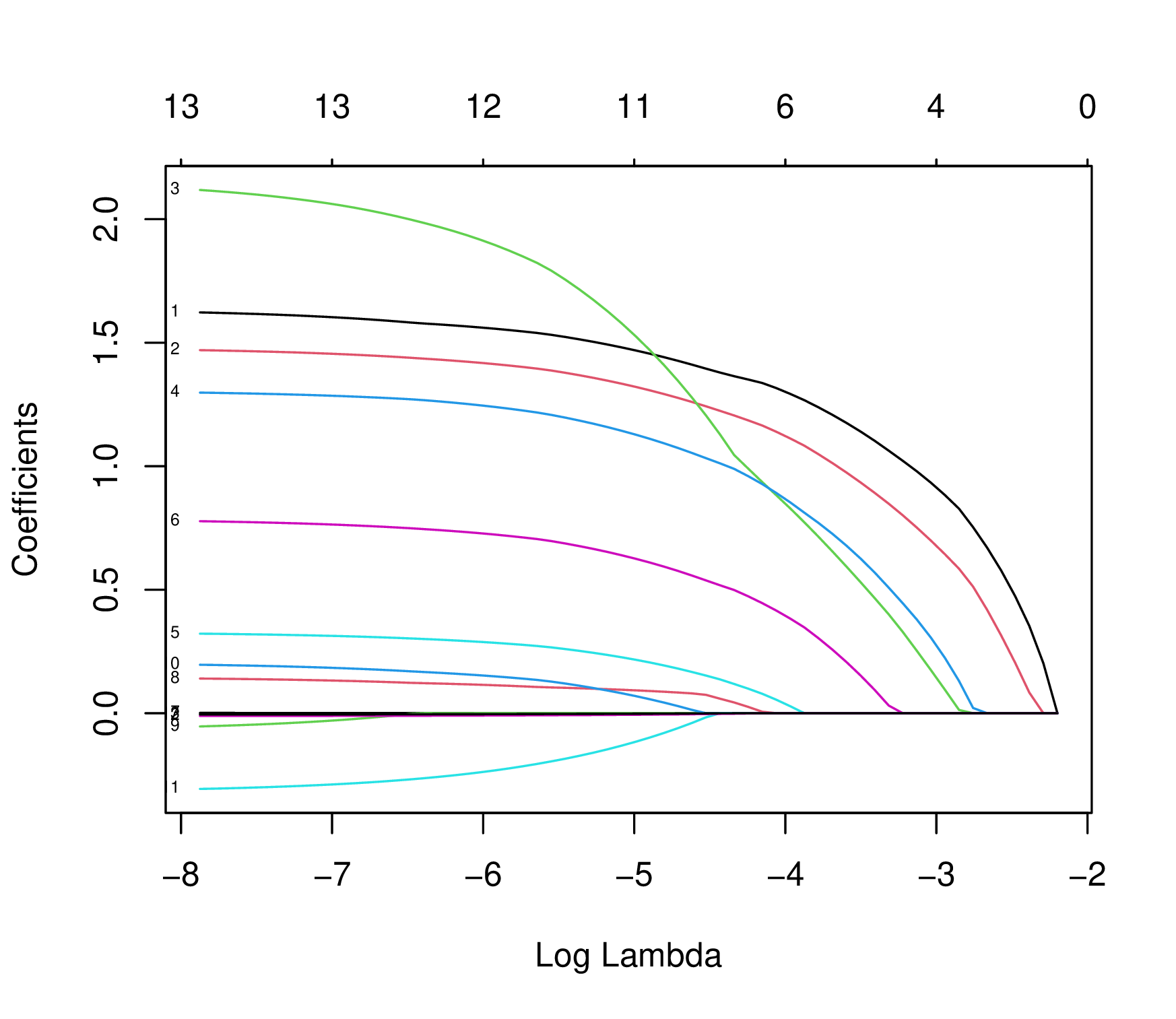

Supplement: Supplemental Information 4 [file peerj-12-18753-s004.png]

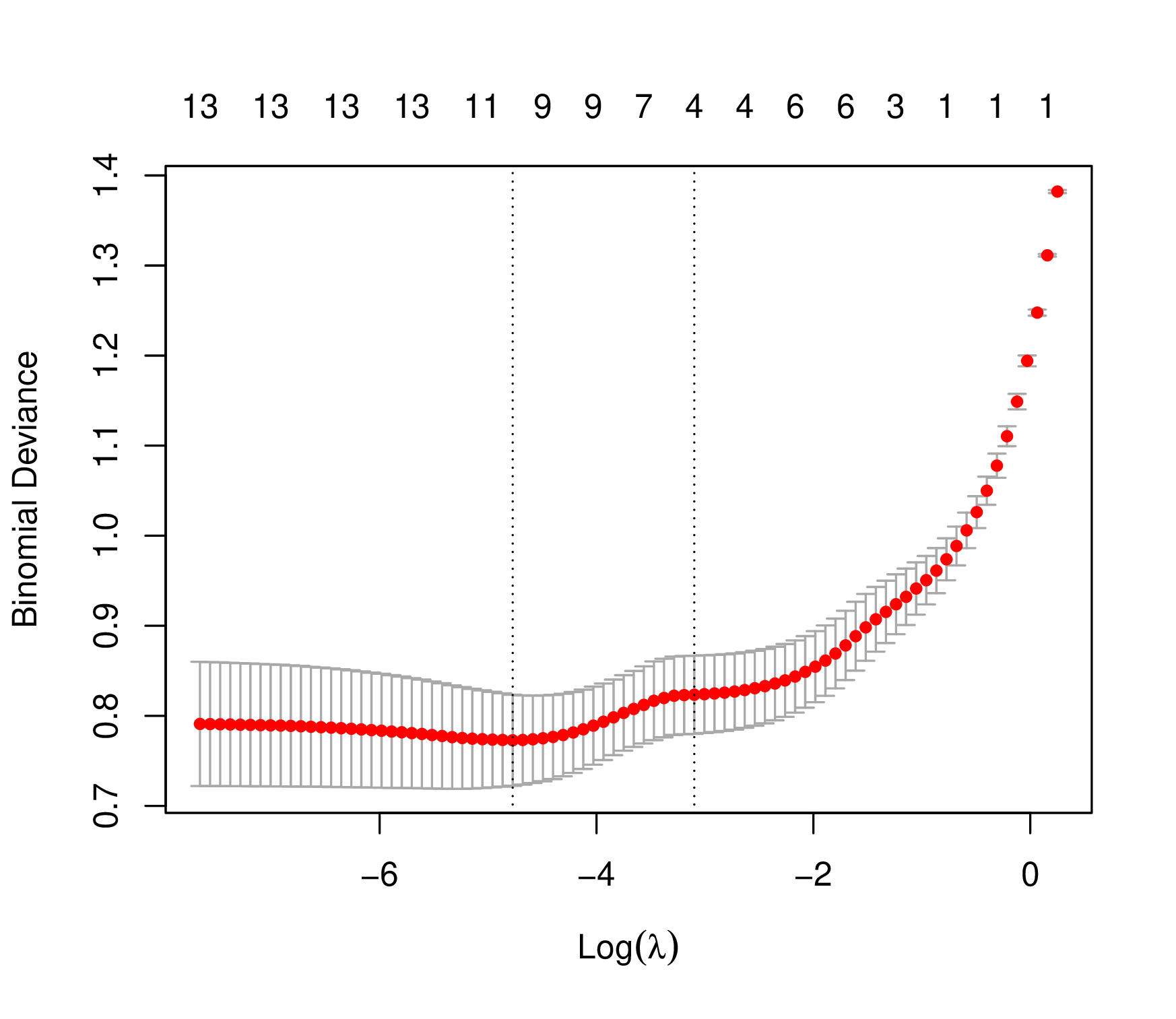

Supplement: Supplemental Information 5 [file peerj-12-18753-s005.png]

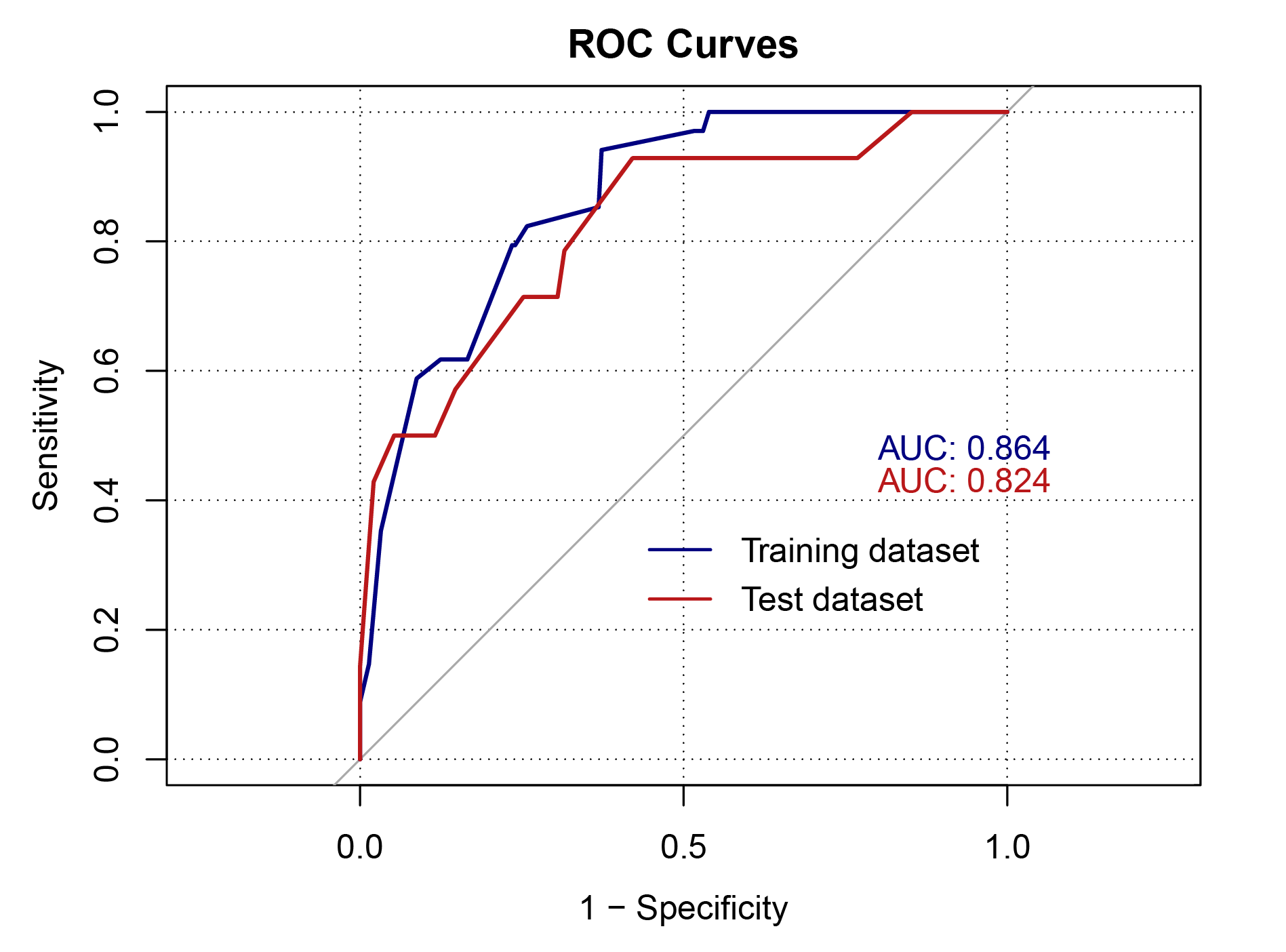

Supplement: Supplemental Information 6 [file peerj-12-18753-s006.png]

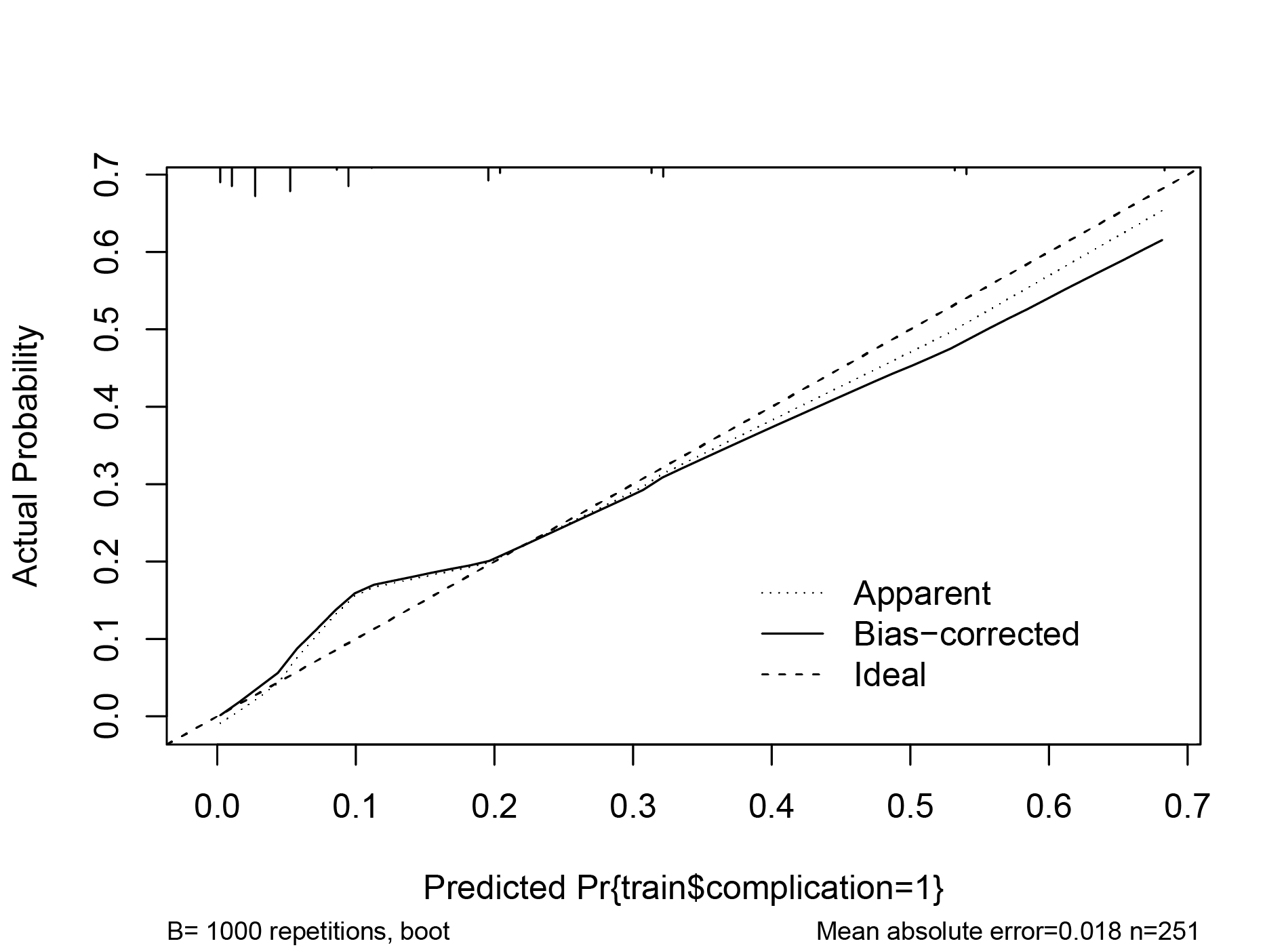

Supplement: Supplemental Information 7 [file peerj-12-18753-s007.png]

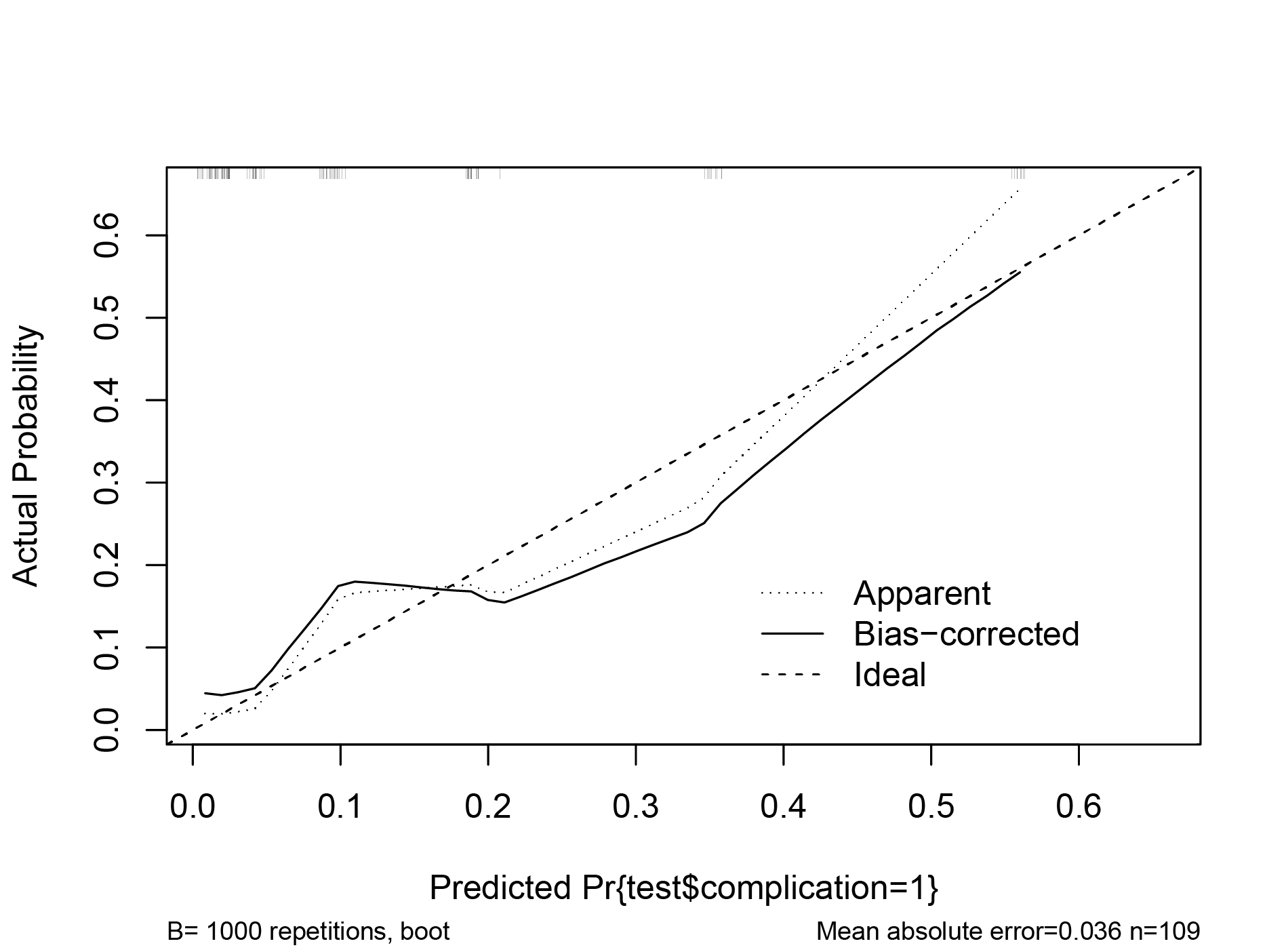

Supplement: Supplemental Information 8 [file peerj-12-18753-s008.png]

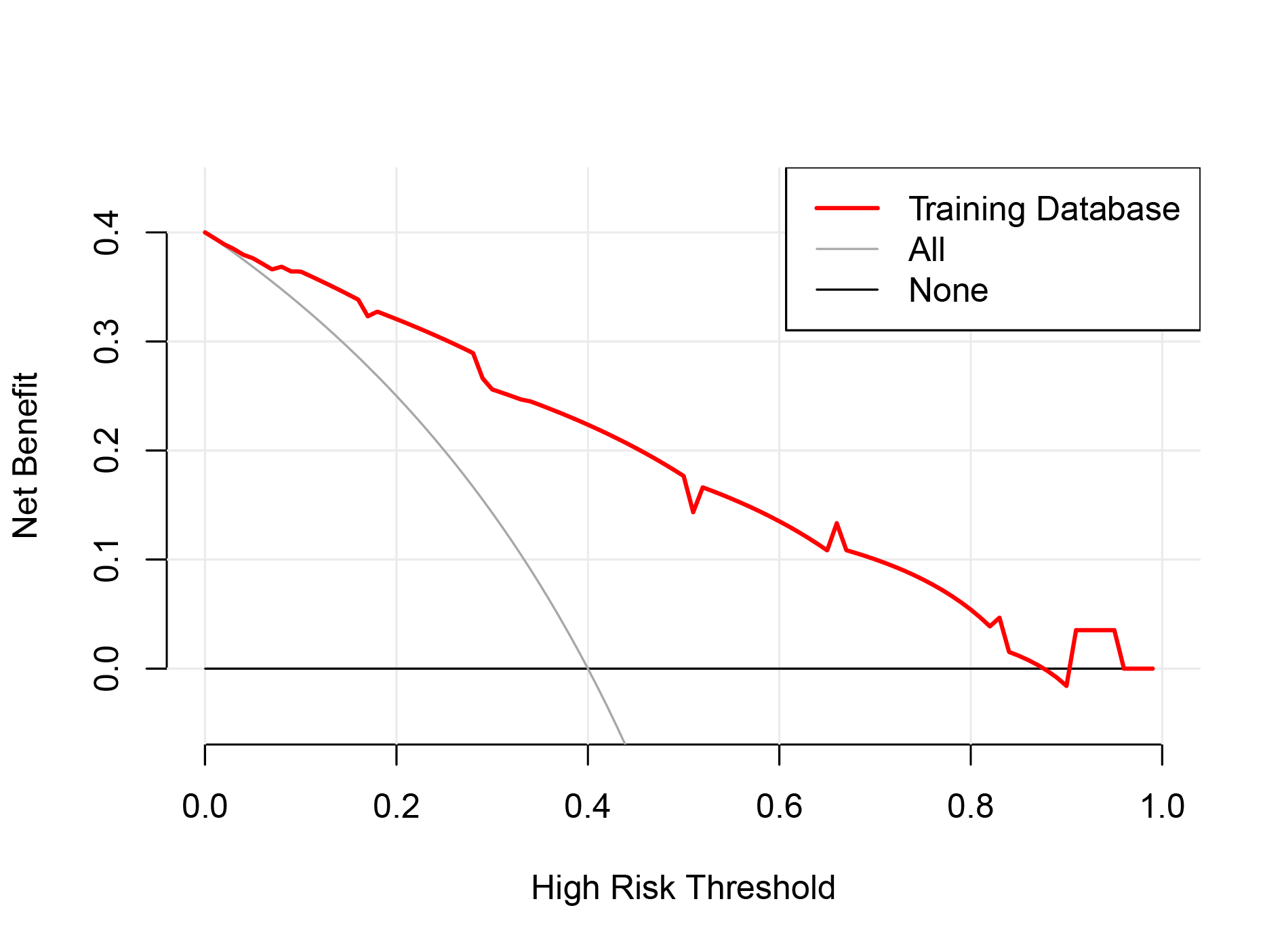

Supplement: Supplemental Information 9 [file peerj-12-18753-s009.png]

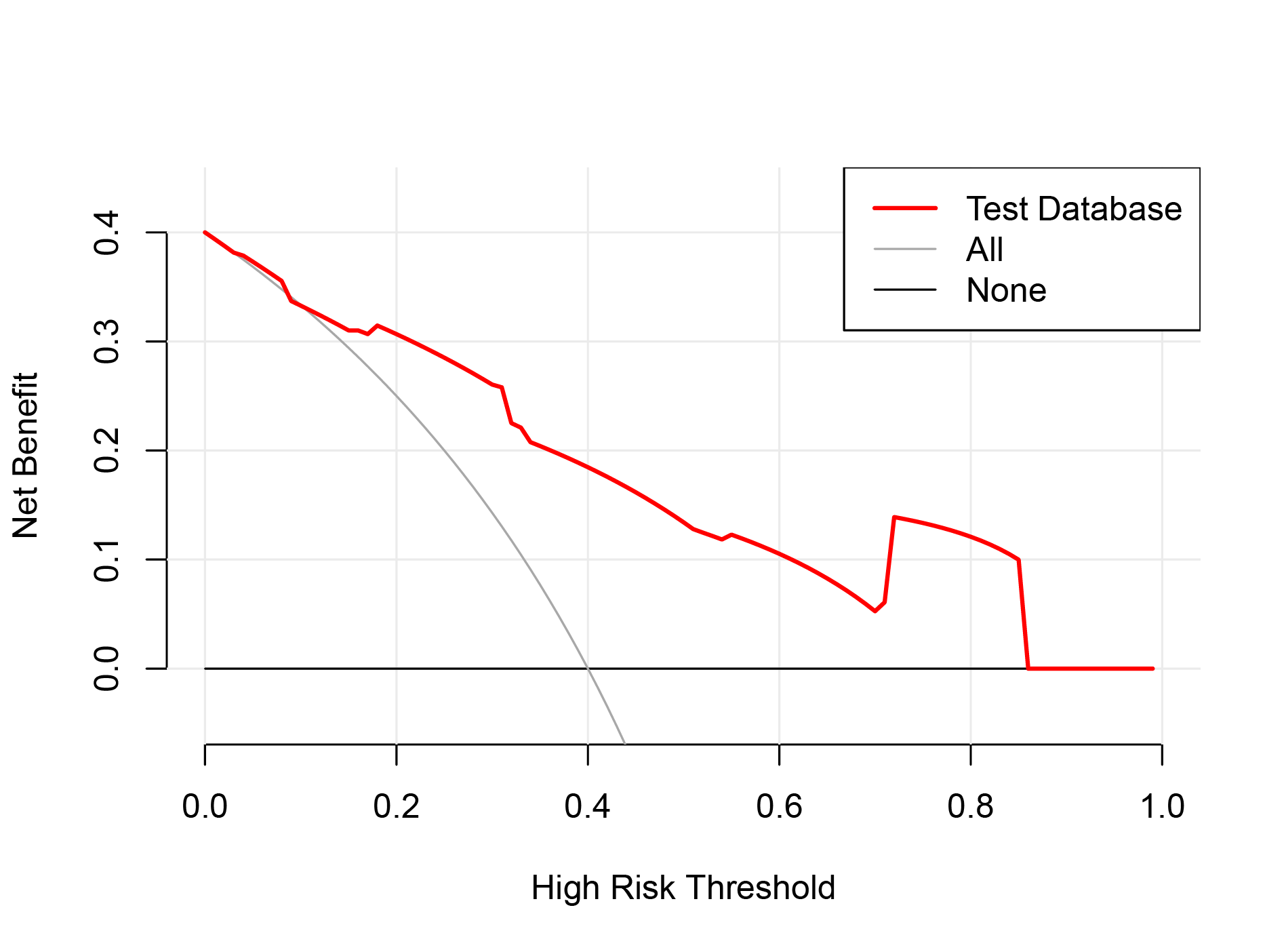

Supplement: Supplemental Information 10 [file peerj-12-18753-s010.png]
