# Supplementary material for: A novel model based on clinical and computed tomography (CT) indices to predict the risk factors of postoperative major complications in patients undergoing pancreaticoduodenectomy
Source: PeerJ. 2024 Dec 19;12:e18753. doi: 10.7717/peerj.18753 (PMC11663404; doi:10.7717/peerj.18753)
Supplement: Supplemental Information 2 [file peerj-12-18753-s002.docx]

Table S1

Distribution of postoperative complications.

| Patients | Number (%) |
| --- | --- |
| Major complications (%) | 48 (13.3) |
| Clavien-Dindo classification (%) |  |
| Without complications | 223 (61.9) |
| I | 48 (13.3) |
| II | 41 (11.4) |
| IIIa | 30 (8.3) |
| IIIb | 8 (2.2) |
| IVa | 6 (1.7) |
| IVb | 4 (1.1) |
