# Supplementary material for: A novel model based on clinical and computed tomography (CT) indices to predict the risk factors of postoperative major complications in patients undergoing pancreaticoduodenectomy
Source: PeerJ. 2024 Dec 19;12:e18753. doi: 10.7717/peerj.18753 (PMC11663404; doi:10.7717/peerj.18753)
Supplement: Supplemental Information 3 [file peerj-12-18753-s003.docx]

Table S2

The incidence of postoperative complications of Clavien-Dindo.

| Complications | Total | Grade I | Grade II | Grade IIIa | Grade IIIb | Grade IVa | Grade IVb |
| --- | --- | --- | --- | --- | --- | --- | --- |
| Pancreatic fistula | 53 | 20 | 18 | 10 | 3 | 1 | 1 |
| Bile leakage | 16 | 5 | 5 | 4 | 2 | 0 | 0 |
| Intestinal fistula | 6 | 0 | 1 | 3 | 1 | 1 | 0 |
| Abdominal infection | 51 | 8 | 21 | 15 | 5 | 1 | 1 |
| Pulmonary infection | 25 | 9 | 8 | 6 | 1 | 1 | 0 |
| PPH | 28 | 4 | 3 | 7 | 13 | 1 | 0 |
| DGE | 33 | 11 | 12 | 8 | 2 | 0 | 0 |
| Organ dysfunction | 9 | 0 | 0 | 0 | 0 | 6 | 3 |

PPH, postpancreatectomy hemorrhage; DGE, delayed gastric emptying.
